# Claims-based algorithm to estimate the Expanded Disability Status Scale for multiple sclerosis in a German health insurance fund: a validation study using patient medical records

**DOI:** 10.3389/fneur.2023.1253557

**Published:** 2023-12-07

**Authors:** Erwan Muros-Le Rouzic, Marco Ghiani, Evi Zhuleku, Anja Dillenseger, Ulf Maywald, Thomas Wilke, Tjalf Ziemssen, Licinio Craveiro

**Affiliations:** ^1^F. Hoffmann-La Roche Ltd., Basel, Switzerland; ^2^IPAM, Institut für Pharmakoökonomie und Arzneimittellogistik e.V., Wismar, Germany; ^3^Cytel Inc., Berlin, Germany; ^4^ZKN, Zentrum für Klinische Neurowissenschaften, Neurologische Klinik, Universitätsklinikum Carl Gustav Carus, Technische Universität Dresden, Dresden, Germany; ^5^AOK PLUS, Dresden, Germany

**Keywords:** multiple sclerosis, Expanded Disability Status Scale, linked-database, medical records, administrative claims data

## Abstract

**Background:**

The Expanded Disability Status Scale (EDSS) quantifies disability and measures disease progression in multiple sclerosis (MS), however is not available in administrative claims databases.

**Objectives:**

To develop a claims-based algorithm for deriving EDSS and validate it against a clinical dataset capturing true EDSS values from medical records.

**Methods:**

We built a unique linked dataset combining claims data from the German AOK PLUS sickness fund and medical records from the Multiple Sclerosis Management System 3D (MSDS^3D^). Data were deterministically linked based on insurance numbers. We used 69 MS-related diagnostic indicators recorded with ICD-10-GM codes within 3 months before and after recorded true EDSS measures to estimate a claims-based EDSS proxy (pEDSS). Predictive performance of the pEDSS was assessed as an eight-fold (EDSS 1.0–7.0, ≥8.0), three-fold (EDSS 1.0–3.0, 4.0–5.0, ≥6.0), and binary classifier (EDSS <6.0, ≥6.0). For each classifier, predictive performance measures were determined, and overall performance was summarized using a macro F1-score. Finally, we implemented the algorithm to determine pEDSS among an overall cohort of patients with MS in AOK PLUS, who were alive and insured 12 months prior to and after index diagnosis.

**Results:**

We recruited 100 people with MS insured by AOK PLUS who had ≥1 EDSS measure in MSDS^3D^ between 01/10/2015 and 30/06/2019 (620 measurements overall). Patients had a mean rescaled EDSS of 3.2 and pEDSS of 3.0. The pEDSS deviated from the true EDSS by 1.2 points, resulting in a mean squared error of prediction of 2.6. For the eight-fold classifier, the macro F1-score of 0.25 indicated low overall predictive performance. Broader severity groupings were better performing, with the three-fold and binary classifiers for severe disability achieving a F1-score of 0.68 and 0.84, respectively. In the overall AOK PLUS cohort (3,756 patients, 71.9% female, mean 51.9 years), older patients, patients with progressive forms of MS and those with higher comorbidity burden showed higher pEDSS.

**Conclusion:**

Generally, EDSS was underestimated by the algorithm as mild-to-moderate symptoms were poorly captured in claims across all functional systems. While the proxy-based approach using claims data may not allow for granular description of MS disability, broader severity groupings show good predictive performance.

## 1. Introduction

Multiple sclerosis (MS) is a chronic immune-mediated disease of the central nervous system and is characterized by inflammation, demyelination, gliosis, and axonal destruction, which lead to the accrual of neurological disability (1). The most common measure of disability in MS is the Expanded Disability Status Scale (EDSS). Originally developed by Kurtzke (2, 3), it is a clinician-rated instrument based on the standard neurological examination of seven functional systems (FS; visual, brainstem, pyramidal, cerebellar, sensory, bowel/bladder and cerebral), and an evaluation of the maximal walking distance without rest (ambulation score). The combined scoring of functional systems and ambulation produces an ordinal scale from 0.0 (normal neurological exam) to 10.0 (death due to MS) with 0.5 increments interval after 1.0 (4). Although well-known limitations such as suboptimal intra- and interrater reliability, non-linearity, marginal sensitivity to change and bias to locomotor function have been described, the EDSS remains the gold standard to classify disability level and worsening in clinical trials (5).

While the EDSS is widely used in clinical trials, it is typically not available in most electronic health records (EHR) or administrative claims databases. This is a major challenge to real-world studies in MS relying on EHR or claims data, especially for the analysis of treatment patterns or related clinical and health-economic outcomes, as information on MS severity and disability level is essential to account for potential confounding and other biases. The estimation of disease severity using claims data is challenging due to missing severity measures (6–8). Administrative claims data provide a detailed comorbidity record and full capture of health care resource use and costs, however clinical information of disease severity is best captured in patient medical records or disease-specific registries. Quantifying the level of disability and disease progression among patients with MS observed in claims databases may improve real-world evidence research in MS, including the investigation of long-term benefits and risks of therapeutic options, optimal treatment utilization, disease behavior, as well as economic and cost-benefit evaluations (9).

In recent years, several studies have proposed approaches to estimate disability levels using claims or EHR data, consisting of algorithms that ranged from expert-led code mapping (6–8, 10–12), regression and machine learning models (13, 14), to more sophisticated deep learning-based natural language processing methods (15). However, only two studies used clinician-recorded EDSS scores as the reference standard for validation of the algorithms (12, 13), with model features derived from unstructured clinical notes, or indicators based on use of particular health care services, specific diagnostic codes, and codes based on employment or social security allowances. Unfortunately, these approaches may not be generalizable to all claims databases depending on data availability.

This study aimed to (1) develop an administrative claims-based proxy EDSS (pEDSS) using a comprehensive list of MS symptoms, treatments, as well as aids and remedies, (2) validate the algorithm against clinician-recorded EDSS scores obtained from a tertiary MS center in Germany, and (3) implement the algorithm to determine pEDSS among patients with MS in a large German sickness fund.

## 2. Materials and methods

### 2.1. Setting

This retrospective cohort study used administrative claims data from a German statutory health insurance (AOK PLUS) linked to medical records from the Multiple Sclerosis Management System 3D (MSDS^3D^), a computer-based patient management system provided by the Center for Clinical Neuroscience in Dresden (Zentrum für Klinische Neurowissenschaften, ZKN) (16–19).

### 2.2. Data sources and linkage

AOK PLUS covers data on all healthcare related services on approximately 3.4 million insured patients in the regions of Saxony/Thuringia in Germany, capturing both inpatient and outpatient settings including hospital admissions, visits to general practitioners and specialists, outpatient prescriptions, rehabilitation stays, as well as aids and remedies. Due to direct relevance for reimbursement, the validity of recording and coding is considered high in claims data, serving as common sources for health-economic and real-world evidence studies (20, 21).

MSDS is an online software which was constructed for better documentation and management of patients with MS, first designed for MS outpatient settings (MSDS Clinic) and later adapted specifically for neurology practices (MSDS Practice) (18, 22, 23). With it's latest development as MSDS^3D^ in 2010 by the MSDS project group in Dresden, the system integrates information from the patient, nurses, and physicians, and supports with more complex activities such as disease management (16, 17, 24). MSDS^3D^ holds patient personal and clinical information for all MS patients followed at ZKN, including administrative data, clinical history, treatment details, disease severity including EDSS scores and functional performance tests.

To generate a linked dataset between AOK PLUS and MSDS^3D^, patients attending regular clinical visits at ZKN who were insured by AOK PLUS were recruited and asked to provide informed consent (19). A list of pseudonymized registry IDs and AOK PLUS insurance numbers were provided by ZKN to AOK PLUS. The registry ID was mapped to a pseudonymized claims data ID using the AOK PLUS insurance numbers, which were subsequently deleted. A linked dataset was generated including pseudonymized registry and claims data IDs, EDSS scores and functional system sub-scores (FSS) with associated date of measurement, date of MS diagnosis, and MS subtype from MSDS^3D^ as well as birth year, sex, insurance coverage, inpatient and outpatient diagnoses (International Classification of Diseases 10th revision, German Modification, ICD-10-GM) and procedures (OPS & EBM, respectively), outpatient prescriptions (ATC), remedies and aids, and date of death from claims data. The dataset was accessible for analysis via the university affiliate IPAM e.V. (Institut für Pharmakoökonomie und Arzneimittellogistik e.V.), who had no access to insurance numbers or other personal data.

### 2.3. Algorithm development and validation study

#### 2.3.1. Development of the proxy EDSS

The development of the claims-based pEDSS was done through multiple iterative steps, with expert input from neurologists with specialization in MS. As the basis for the pEDSS development, the Kurtzke original scale interpretation was used (2, 3), to align with the methodological approach used at ZKN for validation. In the first step, clinical descriptors from the seven FS of the EDSS (i.e., cerebral, visual, sensory, bowel and bladder, pyramidal, cerebellar, or brainstem) were used to search for corresponding MS-related symptoms in the claims database recorded under ICD-10-GM diagnosis codes. For example, “moderate nystagmus and/or moderate extraocular movements impairment” was mapped to the following ICD-10-GM codes: (H49) Paralytic strabismus of oculomotor nerve/trochlear nerve/abducens nerve/unspecified, (H51) Other disorders of binocular movement (H53.278), Diplopia, and (H55) Nystagmus. Overall, 69 MS-related symptoms and corresponding ICD-10-GM codes were identified from claims data (Supplementary Table 1). Some of these codes were also used to determine ambulation status [e.g. (G82.12) Paraparesis and paraplegia, spastic: chronic complete paraplegia]. Alternatively, ambulation was ascertained by identifying potential walking aids (walking sticks, wheelchair, and specialty chair bed) via aids codes (Hilfsmitttel codes, Table 1).

**Table 1:** Claims-based algorithm for computing the EDSS proxy (pEDSS).

**pEDSS step**	**Explanation**	**Definition (ICD-10-GM codes, aids, ATC codes)**
0.0	Normal neurological examination	No diagnosis^a^ of a MS-related symptom^b^ AND No prescription of a MS-related symptom medication or treatment^b^ AND No additional conditions specified for scores >0
1.0	No disability	At least one of the following conditions: • Diagnosis of 1 mild MS-related symptom • A prescription of a drug/therapy associated with a mild MS-related symptom AND No prescription of fampridine (ATC: N07XX07) AND No additional condition specified for scores >1
2.0	Minimal disability	Diagnosis^a^ of ≥2 mild MS-related symptoms^b^ AND No prescription of fampridine (ATC: N07XX07) AND No additional condition specified for scores >2
3.0	Moderate disability	At least one of the following conditions: • Diagnosis of ≤ 2 moderate MS-related symptoms^b^ (except gait disturbance) • Prescription of ≤ 2 drugs/therapies associated with a moderate MS-related symptom AND No prescription of fampridine (ATC: N07XX07) AND No additional condition specified for scores >3
4.0	Relatively severe disability	At least one of the following conditions: • Diagnosis^a^ of 3 moderate MS-related symptoms^b^ • Prescription of at least 3 drugs/therapies associated with a moderate MS-related symptom^b^ • Gait disturbance (ICD-10-GM: R26.0, R26.1, R26.2, R26.8) • Prescription of fampridine (ATC: N07XX07) with no diagnoses of moderate or severe symptoms^b^ AND No additional condition specified for scores > 4
5.0	Disability precludes fill daily activities (with potentially more impaired ambulation)	At least one of the following conditions: • Diagnosis^a^ of ≥1 severe MS-related symptom^b^ • Prescription of ≥1 drug/therapy associated with a severe MS-related symptom^b^ • Diagnosis^a^ of ≥4 moderate MS-related symptoms^b^ • Prescription of fampridine (ATC: N07XX07) with diagnosis of at least one moderate or severe symptom^b^ AND No additional condition specified for scores > 5
6.0	Assistance required to walk	Walking stick required (aids code: 10.50.01) AND No additional condition specified for scores > 6
7.0	Restricted to wheelchair	Wheelchair required (aids codes: 18.50 and 18.51) OR diagnosis^a^ of paraplegia (ICD-10-GM: G82.12, G82.22, G82.63, M62.3) AND No additional condition specified for scores > 7
8.0	Restricted to bed or chair	Chair or specialty bed (aids code: 19.40.01) required OR diagnosis^a^ of tetraplegia (ICD-10-GM: G82.42, G82.52) AND No additional condition specified for scores > 8
9.0	Confined to bed	Confined to bed (ICD-10-GM: R26.3) AND Alive by end of the study period

a Diagnosis refers to at least one inpatient and/or two confirmed outpatient diagnoses.

b Symptoms, related medication or treatment, and categorization into mild, moderate, and severe are defined in the Supplementary Table 1.

In the second step, an assessment of symptom severity (i.e., mild, moderate, or severe) was conducted based on the impact of symptoms on the EDSS calculation (e.g., although fatigue and depression have a high impact on quality of life, the cerebral FSS has a reduced contribution to the EDSS calculation), and/or the type of treatment used to manage the MS-related symptom (e.g., mild spasticity if only the clinical descriptor was identified, moderate if clinical descriptor + pharmacological treatment (e.g. baclofen), and severe if clinical descriptor + interventional treatment (e.g. intrathecal baclofen pump). Additional details can be found in Supplementary Table 1. A detailed comparison was performed between all questions in the functional system scores recorded in MSDS^3D^ and the symptoms and severity levels derived from the claims (Supplementary Table 2).

In the final step, MS-related symptoms with respective severity assessment, therapies, and aids were mapped to an EDSS level according to the algorithm described in Table 1. While the EDSS provides a total score ranging from 0.0 to 10.0 with twenty possible steps, the pEDSS was developed to predict a score with the same range but only 10 possible steps (Table 1). The algorithm was truncated to exclude the EDSS step of 10.0 (death) because our goal was to predict disability status among living individuals.

#### 2.3.2. Validation study of the pEDSS

##### 2.3.2.1. Analytical approach

**Figure 1:**
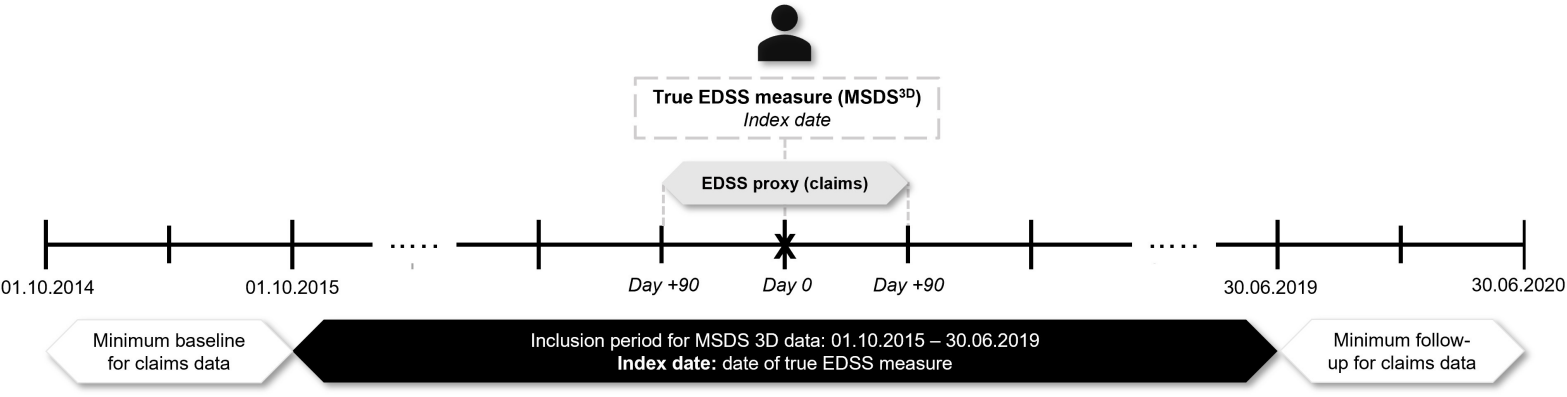
Validation study design.

Patients with an MS diagnosis enrolled in AOK PLUS with ≥1 true measure of the EDSS score in MSDS^3D^ between 01/10/2015 and 30/06/2019 were selected. The date of the first observable EDSS recording in MSDS^3D^ was set as the index date. The pEDSS was computed using claims data within a window of 3 months before and after each index date. As such, patients were required to be continuously insured for ≥3 months before and after the index date (Figure 1). In a sensitivity approach, pEDSS was calculated based on all true EDSS scores recorded in MSDS^3D^ during the available follow-up period, with each patient able to contribute more than one EDSS/pEDSS value.

As described above, the pEDSS was built as an ordinal scale of 0 to 9 with only 10 possible steps. Given that the EDSS includes 20 possible steps in increments of 0.5 points, we first rescaled the EDSS scores from MSDS^3D^ as follows: (1) from EDSS 1.0 to 7.5, all half-point scores were converted to the lower step (e.g., EDSS scores of 2.5 and 2.0 were rescaled as 2.0); (2) from EDSS 8.0 to 9.5, scores were grouped as ≥8.0, as these scores reflect the same construct of daily living activity on patients without any ambulation. Moreover, a low number of patients within this EDSS range were available from the MSDS^3D^ validation cohort. Finally, given that no patients with an EDSS score of 0.0 (normal neurological examination) were available in the MSDS^3D^ validation cohort, the pEDSS scores of 0.0 were imputed as pEDSS of 1.0.

##### 2.3.2.2. Model classifiers

Our primary goal was to develop an eight-fold classifier model which would predict each EDSS step from 1.0 to 7.0 and the aggregate of scores ≥8.0 (excluding 10.0). Two alternative models with broader classifications were also tested, including a three-fold classifier for the categories EDSS 1.0–3.0, EDSS 4.0–5.0 and EDSS ≥6.0, and a binary classifier for EDSS <6.0 vs. EDSS ≥6.0. These categories were chosen because they represent clinically relevant classifications (4).

##### 2.3.2.3. Performance metrics

Multi-class confusion matrices were built for the different classifiers, with information on true positives (TP), true negatives (TN), false positives (FP) and false negatives (FN). From the confusion matrix the following performance metrics were calculated for each class: Sensitivity also referred to as recall (TP/[TP+FN]), Specificity (TN/[FP+TN]), Positive predictive value (PPV) also referred to as precision (TP/[TP+FP]), Negative predictive value (NPV) defined as (TN/[FN+TN]), Accuracy, which is the ratio of correct predictions made (TP+TN) to the total number of predictions made (TP+TN+FP+FN), Cohen's kappa coefficient (K) to assess the degree of agreement, and finally the F1-score which is a metric that combines precision and recall into a single number using the harmonic mean, and provides a more robust measure of incorrectly classified cases in imbalanced class settings. The overall performance of pEDSS model classifiers was evaluated using a macro-averaged F1-score (or macro F1-score) which is computed using the arithmetic mean of F1-scores of all respective classes.

Finally, mean EDSS and pEDSS were calculated for the validation cohort at index and across all measures over the study period. The overall performance of the model was summarized using the mean-squared error (MSE).

Demographic and clinical characteristics of the study cohorts were summarized using descriptive statistics including mean, standard deviation (SD), median, range (minimum, maximum) and frequency (percent) as applicable. Statistical analyses were performed using STATA 17 (StataCorp. 2021. Stata Statistical Software: Release 17. College Station, TX: StataCorp LLC).

#### 2.3.3. Implementation of the pEDSS in the AOK PLUS population

The claims-based pEDSS algorithm was finally implemented across the entire MS population in the AOK PLUS dataset. Patients with ≥1 inpatient or ≥2 confirmed outpatient diagnoses from a neurologist (ICD-10-GM: G35.-) in the inclusion period between 01/07/2016 and 30/06/2017 were selected. The index date was defined as the date of the first observable MS diagnosis in the inclusion period. Adult patients who were not continuously insured (excluding death) or had diagnoses related to pregnancy or demyelinating disease between 12 months before and after the index to 30/06/2018 were excluded, allowing for a baseline and follow-up period of 12 months before and after the index date, respectively, whereby pEDSS was computed. As the algorithm was designed to predict disability among living patients, we further filtered out patients that died in the 12-month follow-up. Disability levels using the pEDSS were calculated according to multiple age strata (18–50, 51–65, >65 years), sex (female, male), type of MS (RRMS, progressive MS [PMS] comprising SPMS and PPMS, unspecified) and presence of comorbidities (0, 1, ≥2). The methodology for identifying patient MS subtypes and the list of comorbidities used in this study have been previously described (25).

## 3. Results

### 3.1. Patient characteristics and EDSS distribution

Overall, 100 patients with MS with ≥1 EDSS score recorded in MSDS^3D^ were included in the analysis. Demographic and clinical characteristics at baseline are presented in Table 2. The study sample was representative of a typical MS population, with 75.0% female patients, an overall mean age (SD) of 48.3 (12.7) years and the majority of patients (74.0%) classified as having relapsing-remitting MS (RRMS).

**Table 2:** Baseline characteristics at the index EDSS measurement in the study period.

**Patient Characteristics**	**Patients with MS *N* = 100**
**Demographics**	
Age at index, mean years (SD)	48.3 (12.7)
Female, *n* (%)	75 (75.0)
**Clinical characteristics**	
Time since initial diagnosis^a^, mean years (SD)	10.0 (9.0)
MS subtype, *n* (%)
RRMS	74 (74.0)
PPMS	4 (4.0)
SPMS	14 (14.0)
ND/CIS	8 (8.0)
Year of index, *n* (%)
2015	24 (24.0)
2016	21 (21.0)
2017	17 (17.0)
2018	26 (26.0)
2019	12 (12.0)
Number of EDSS scores per patient in study period, mean (SD)	6.2 (5.1)
EDSS at index, mean (SD)	3.4 (1.8)
Rescaled EDSS at index^b^, mean (SD)	3.2 (1.8)
EDSS at index by category, *n* (%)
1.0–1.5	18 (18.0)
2.0–2.5	23 (23.0)
3.0–3.5	27 (27.0)
4.0–4.5	11 (11.0)
5.0–5.5	4 (4.0)
6.0–6.5	9 (9.0)
7.0–7.5	7 (7.0)
≥8.0	1 (1.0)
Functional system scores at index, mean (SD)
Visual (ordinal range 0–6)	1.1 (1.0)
Brainstem (ordinal range 0–5)	0.9 (0.8)
Pyramidal (ordinal range 0–6)	1.9 (1.1)
Cerebellar (ordinal range 0–5)	1.4 (1.1)
Sensory (ordinal range 0–6)	1.6 (1.1)
Bowel and bladder (ordinal range 0–6)	0.9 (1.0)
Cerebral (ordinal range 0–5)	1.1 (0.9)
Ambulation (ordinal range 0–12)	1.9 (3.3)

a Date of initial diagnosis as recorded in MSDS3D, missing for 7 patients.

b From EDSS 1.0 to 7.5, all half-point scores were converted to the lower step (e.g., EDSS scores of 2.5 and 2.0 were rescaled as 2.0).

CIS, clinically isolated syndrome; EDSS, Expanded Disability Status Scale; MS, multiple sclerosis; ND, newly diagnosed; PPMS, primary progressive multiple sclerosis; RRMS, relapsing-remitting multiple sclerosis; SD, standard deviation; SPMS, secondary progressive multiple sclerosis.

A total of 620 EDSS scores were available across all patients, ranging from 1 to 18 scores per patient (mean 6.2 EDSS). Half of the patient population had an EDSS between 2.0 and 3.0 (23.0% with 2.0–2.5, 27.0% with 3.0–3.5), and only one patient had a score ≥8.0 (1.0%).

The mean EDSS at index was 3.4 before and 3.2 after rescaling of the 0.5-increments (Table 2). The mean FSS ranged from 0.9 for brainstem to 1.9 for bowel and bladder, with a mean 1.9 points additionally captured by ambulation.

### 3.2. Validation of pEDSS vs. true EDSS

#### 3.2.1. Mean observed EDSS vs. mean pEDSS

**Figure 2:**
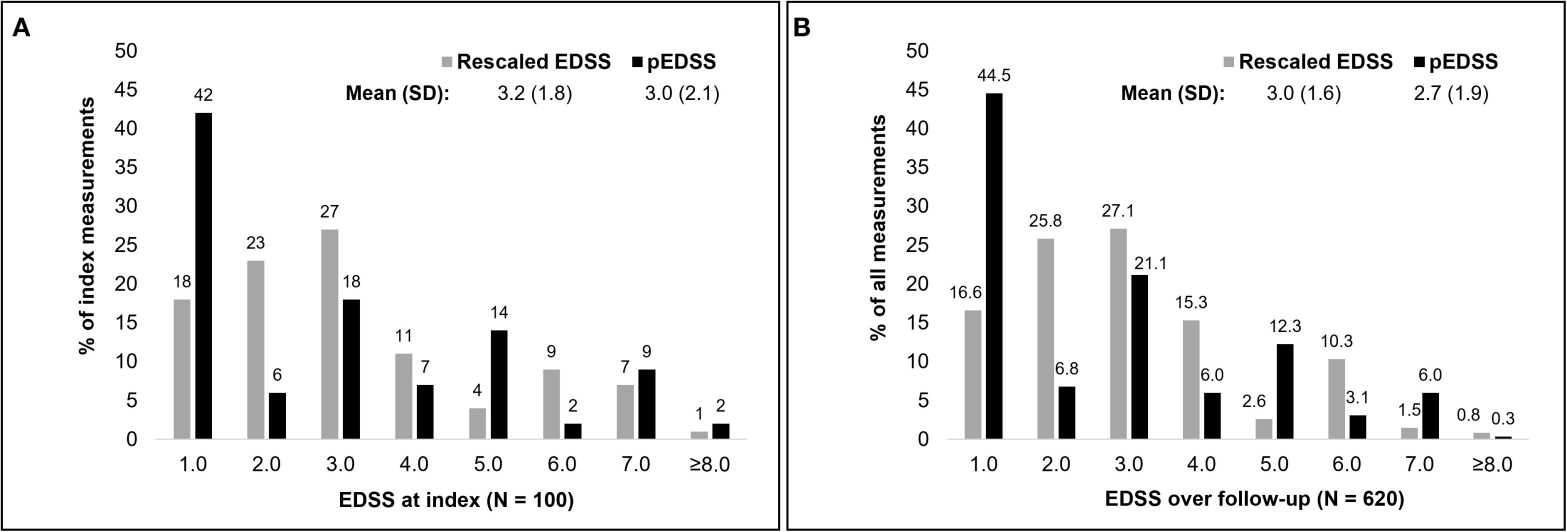
Distribution of the rescaled EDSS and claims based pEDSS at index EDSS assessment **(A)** and across all EDSS in the follow-up **(B)**.

Upon derivation of the pEDSS using the algorithm outlined in Table 1, the mean (SD) pEDSS was 3.0 (2.1), compared to a mean 3.4 (1.8) true EDSS and 3.2 (1.8) rescaled EDSS of the index (first) EDSS measures in MSDS^3D^ (Figure 2). The index pEDSS deviated from the rescaled EDSS by 1.2 points (mean absolute error). Across all 620 observed EDSS measures, mean (SD) pEDSS was 2.7 (1.9) compared to 3.2 (1.5) true EDSS and 3.0 (1.6) rescaled EDSS. The MSE of prediction of the index pEDSS was 2.6, compared to MSE of 2.5 when evaluating all 620 available measures in the patient follow-up.

Within the validation patient sample, mean rescaled EDSS and pEDSS at index were highest among patients with PMS [rescaled EDSS 5.5 (1.5); pEDSS 5.3 (2.1)], followed by patients aged ≥50 years [rescaled EDSS 4.3 (1.8), pEDSS 3.9 (2.2)] (Table 3). Among all subgroups, differences in mean rescaled EDSS and pEDSS at index were not statistically significant.

**Table 3:** Mean (SD) EDSS and pEDSS at index according to key patient characteristics.

		**Index EDSS (N** = **100)**				**All EDSS in the follow-up (N** = **620)**			
		***N***	**Rescaled EDSS mean (SD)**	**pEDSS mean (SD)**	***P*** **-value**	***N***	**Rescaled EDSS mean (SD)**	**pEDSS mean (SD)**	***P*** **-value**
MS type	RRMS/CIS	82	2.7 (1.5)	2.5 (1.8)	0.163	566^a^	2.8 (1.5)	2.5 (1.8)	0.000
	PMS	18	5.5 (1.5)	5.3 (2.1)	0.708	50^a^	5.2 (1.3)	4.6 (2.0)	0.046
Age	< 50 years	53	2.2 (1.3)	2.2 (1.7)	0.707	370	2.2 (1.0)	2.1 (1.5)	0.101
	≥50 years	47	4.3 (1.8)	3.9 (2.2)	0.126	250	4.2 (1.6)	3.5 (2.1)	0.000
Sex	Female	75	3.3 (2.0)	3.2 (2.2)	0.571	462	3.0 (1.7)	2.7 (1.8)	0.000
	Male	25	2.8 (1.4)	2.2 (1.8)	0.066	158	2.9 (1.4)	2.7 (2.1)	0.062

^a^MS subtype was missing at 4 measurements in the follow-up.

#### 3.2.2. Eight-fold EDSS classifier

Compared to the EDSS observed in MSDS^3D^, the pEDSS showed a tendency to underestimate the true level of disability. Generally, scores 1.0 and 5.0 were overestimated, whereas scores 2.0, 3.0 and 6.0 were underestimated (Figure 2). Based on the observable claims data symptoms, the cerebral FSS was the most documented FSS (27% of patients with ≥1 symptom of any severity), followed by sensory (25%), bowel/bladder (24%), and pyramidal (23%) (Supplementary Table 2). Brainstem symptoms were the least frequently observed in claims data (2%). For most functional systems, a high proportion of patients with mild to moderate FSS (1–3) had no symptoms of any severity recorded in claims data, explaining the underestimation of pEDSS at moderate levels of disability (Supplementary Tables 1, 2). For ambulation, of seven patients with a wheelchair (true ambulation score 10–12), six (86%) had a recorded wheelchair in claims data. Of nine patients requiring unilateral or bilateral assistance, only two (22%) had a documented walking stick aid. No bed confinement codes were observed in claims data, congruent with 0 patients in MSDS^3D^ who were confined to a bed.

Assessing the score-wise performance of the eight-fold classifier, precision was the highest for EDSS 2.0 (0.67) and lowest for EDSS ≥8.0 and 5.0 (0.00 and 0.07, respectively) (Table 4). Sensitivity was highest for EDSS 7.0, where 57.0% who had a rescaled EDSS 7.0 correctly had a pEDSS 7.0. Overall performance of the pEDSS was highest for EDSS 7.0 (F1-score = 0.50) and lowest for scores ≥8.0 (F1-score = 0.00), followed by scores 4.0, 5.0 and 6.0 (F1-score = 0.11, 0.11, 0.18, respectively). Macro F1-score for all EDSS was 0.25 indicating low overall predictive performance.

**Table 4:** Predictive performance of EDSS proxy as eight-fold EDSS classifier, three-fold, and binary severity classifier.

	**TP**	**TN**	**FP**	**FN**	**Sensitivity (Recall)**	**Specificity**	**PPV (Precision)**	**NPV**	**Accuracy**	**Cohen's Kappa**	**F1-score**
**Eight-fold EDSS classifier**											
EDSS 1.0	13	53	29	5	0.72	0.65	0.31	0.91	0.66	0.24	0.43
EDSS 2.0	4	75	2	19	0.17	0.97	0.67	0.80	0.79	0.20	0.28
EDSS 3.0	8	63	10	19	0.30	0.86	0.44	0.77	0.71	0.18	0.36
EDSS 4.0	1	83	6	10	0.09	0.93	0.14	0.89	0.84	0.03	0.11
EDSS 5.0	1	83	13	3	0.25	0.87	0.07	0.97	0.84	0.05	0.11
EDSS 6.0	1	90	1	8	0.11	0.99	0.50	0.92	0.91	0.15	0.18
EDSS 7.0	4	88	5	3	0.57	0.95	0.44	0.97	0.92	0.46	0.50
EDSS ≥8.0	0	97	2	1	0.00	0.98	0.00	0.99	0.97	−0.01	0.00
**Three-fold classifier**											
EDSS 1.0–3.0	58	24	8	10	0.85	0.75	0.88	0.71	0.82	0.59	0.87
EDSS 4.0–5.0	8	72	13	7	0.53	0.85	0.38	0.91	0.80	0.33	0.44
EDSS ≥6.0	11	81	2	6	0.65	0.98	0.85	0.93	0.92	0.69	0.73
**Binary classifier**											
EDSS < 6.0	81	11	6	2	0.98	0.65	0.93	0.85	0.92	0.69	0.95
EDSS ≥6.0	11	81	2	6	0.65	0.98	0.85	0.93	0.92	0.69	0.73

Sensitivity/Recall: TP/(TP+FN); Specificity: TN/(FP+TN); PPV/Precision: TP/(TP+FP); NPV: TN/(FN+TN); Accuracy: (TP+TN)/(TP+TN+FP+FN); F1-Score: harmonic mean of recall and precision.

EDSS, Expanded Disability Status Scale; FN, false negative; FP, false positive; NPV, negative prediction value; PPV, positive prediction value; TN, true negative; TP, true positive.

#### 3.2.3. Three-fold severity classifier

Precision for the three-fold severity classifier was highest for low (EDSS 1.0–3.0) and severe (EDSS ≥6.0) disability groupings with PPV of 87.9% and 84.6%, respectively (Table 4). Sensitivity was highest for low severity, with 85.3% of low severity cases (MSDS^3D^) correctly classified by the EDSS proxy. The overall performance was lowest for moderate disability (EDSS 4.0–5.0), with an F1-score of 0.44, indicating difficulty in predicting moderate levels of disability, consistent with observations from the eight-fold classification. With a macro F1-score of 0.68, the three-fold classifier was an improvement over the eight-fold EDSS classifier.

#### 3.2.4. Binary classifier

The binary classifier of EDSS <6.0 vs. EDSS ≥6.0 showed the best predictive performance, with a precision of 84.6% for predicting EDSS ≥6.0 (Table 4). Almost all scores of EDSS <6.0 were correctly classified, with 97.6% specificity. The overall accuracy for severe disability prediction was 0.92, with a final F1-score of 0.73. Overall, Cohen's K for this binary classifier of was 68.7% (95% Confidence Intervals [CI]: 48.6–88.8) and the macro F1-score was 0.84.

#### 3.2.5. Sensitivity analyses

When computing pEDSS for all 620 EDSS measures in MSDS^3D^ whereby multiple scores were available per patient across the entire available follow-up, we consistently observed similar predictive performance (Supplementary Table 3). In line with index pEDSS results, using all available EDSS, the macro-average F1-score reached was 0.21, 0.61, and 0.80 for the eight-fold, three-fold, and binary classifiers, respectively.

### 3.3. pEDSS in the AOK PLUS cohort

After validation of the algorithm, pEDSS was computed among 3,756 patients with MS (71.9% female, 51.4% with RRMS, mean 51.9 years) in AOK PLUS, alive and continuously insured in the 12-month baseline and follow-up periods before and after index MS diagnosis, respectively. Disability levels using pEDSS in the follow-up stratified by patient subgroups are shown in Table 5 (refer to Supplementary Table 4 for baseline assessment). Overall, disability was most severe among patients with increasing age (mean pEDSS 5.4 for patients >65), PMS diagnosis (mean pEDSS of 5.5 PPMS/SPMS vs. 3.3 RRMS) and increasing number of comorbidities at baseline (mean pEDSS of 2.7, 3.6, and 4.8 for 0, 1, and 2+ comorbidities, respectively). Overall, pEDSS in the baseline and follow-up among the AOK PLUS cohort resulted in a bimodal distribution, peaking at EDSS 1.0–3.0, and 5.0 (Supplementary Figures 1–3).

**Table 5:** pEDSS of the AOK PLUS cohort in the follow-up according to key patient characteristics.

**Category**		***N***	**Overall pEDSS**	**pEDSS**, ***n*** **(%)**			**pEDSS**, ***n*** **(%)**	
			**Mean (SD)**	**0–3.0**	**4.0–5.0**	≥**6.0**	<**6.0**	≥**6.0**
Overall	All	3,756	3.9 (2.2)	1,811 (48.2)	1,366 (36.4)	579 (15.4)	3,177 (84.6)	579 (15.4)
Sex	Female	2,700	3.8 (2.2)	1,332 (49.3)	988 (36.6)	380 (14.1)	2,320 (85.9)	380 (14.1)
	Male	1,056	4.0 (2.4)	479 (45.4)	378 (35.8)	199 (18.8)	857 (81.2)	199 (18.8)
Age (years)	18–50	1,716	3.0 (1.9)	1,144 (66.7)	460 (26.8)	112 (6.5)	1,604 (93.5)	112 (6.5)
	51–65	1,357	4.2 (2.1)	530 (39.1)	596 (43.9)	231 (17.0)	1,126 (83.0)	231 (17.0)
	>65	683	5.4 (2.2)	137 (20.1)	310 (45.4)	236 (34.6)	447 (65.4)	236 (34.6)
Type of MS	RRMS	1,929	3.3 (2.0)	1,139 (59.0)	653 (33.9)	137 (7.1)	1,792 (92.9)	137 (7.1)
	PMS	652	5.5 (2.1)	102 (15.6)	308 (47.2)	242 (37.1)	410 (62.9)	242 (37.1)
	Unspecified	1,175	4.0 (2.3)	570 (48.5)	405 (34.5)	200 (17.0)	975 (83.0)	200 (17.0)
Baseline comorbidities	0	1,056	2.7 (1.9)	762 (72.2)	231 (21.9)	63 (6.0)	993 (94.0)	63 (6.0)
	1	1,019	3.6 (2.0)	535 (52.5)	375 (36.8)	109 (10.7)	910 (89.3)	109 (10.7)
	≥2	1,681	4.8 (2.1)	514 (30.6)	760 (45.2)	407 (24.2)	1,274 (75.8)	407 (24.2)

## 4. Discussion

The EDSS is the gold standard for measuring disability and disease severity in MS and plays an important role in monitoring disease progression and informing clinical decisions (4, 26). However, in real-world data sources, EDSS scores are documented infrequently. In this study, we developed a rule-based algorithm to predict EDSS, using symptoms, medications, and aids recorded in administrative healthcare claims data from a large sickness fund (AOK PLUS) in Germany. The algorithm was then validated against clinician-derived EDSS data obtained from a large, specialized MS care center. While a number of groups have previously attempted to derive EDSS or disability in MS from real-world data, especially claims or electronic medical records (6–8, 10–15), only two previous studies followed a similar validation strategy using a clinical reference standard (i.e., true EDSS) (12, 13).

We built three different models, one that would allow an estimation of single EDSS steps (eight-fold classifier of EDSS scores 1.0–7.0, ≥8.0), and two predicting disability levels according to well-established EDSS categories (three-fold categorical classifier of low, moderate, or severe disability and binary classifier of severe or non-severe disability). When trying to estimate eight different EDSS steps, the pEDSS exhibited overall low precision and low sensitivity, particularly for pEDSS scores of 4.0 and 5.0, but high accuracy. An overestimation of the step 1.0 was also observed, with pEDSS 1.0 calculated for 42% of patients compared to 18% of patients with a true rescaled EDSS 1.0 (MSDS^3D^). This is partly due to the imputation of pEDSS 0.0 values as pEDSS 1.0, given that EDSS 0.0 was not recorded for any of the patients in the MSDS^3D^ dataset. Overall, these observations suggest that mild to moderate symptoms and respective treatments are poorly captured in claims data, potentially reflecting low relevance for reimbursement purposes. In contrast, such symptoms are likely to be recorded in the medical records. The pEDSS 7.0 had the highest F1-score (0.50) reflecting the more complete recording of severe indicators in claims data, such as symptoms requiring immediate medical attention and ambulatory aids relevant for reimbursement (6 of 7 true patients with a wheelchair were captured). However, the pEDSS 6.0 had a very low F1-score (0.18), which resulted from use of walking sticks not being captured for all patients requiring unilateral or bilateral assistance, potentially due to relative accessibility of such aids outside of the insurance reimbursement system.

Similar to our work, two previous studies attempted to derive multiple EDSS steps from real-world sources. One study in Canada linked administrative data to a large clinical dataset to develop a regression-based algorithm that predicted the EDSS (including 0.5 increments) as a continuous measure. The best performing model explained 40% of EDSS variation (pseudo-R^2^ 0.40) with a MSE of 2.09, which is consistent with the MSE of 2.6 observed for our algorithm (12). Our work affirms the challenges with deriving a granular EDSS proxy, largely attributed to claims data coding practices of relevant signs and symptoms of lower severity. Another study used a natural language processing model that combined a rule-based approach with a deep learning model for extracting and/or deriving EDSS scores from the records of patients with MS. In almost two thirds of cases, the model worked by extracting the exact EDSS score annotation and, not surprisingly, this resulted in a macro F1-score of 0.90. However, when the same model was applied to the clinical notes without an explicit EDSS score, the performance was much lower with a macro F1-score of 0.39, which is similarly low to the macro F1-score of 0.25 observed for our eight-fold model (15).

When using the algorithm to estimate broader EDSS categories, we observed that EDSS scores 4.0 and 5.0 were poorly classified and underestimated (precision/PPV 0.38, F1-score 0.44), whereas EDSS scores 1.0–3.0 and EDSS ≥6.0 showed higher precision, with more than 85% of cases correctly predicted. The lower precision observed for pEDSS 4.0–5.0, can be partly explained by the non-linear properties and the bimodal distribution of EDSS, which is reflected by patients staying for the shortest time in the middle scores (4.0–5.0) and peaks at 1.0–3.0 and 6.0–7.0 (27). As PPV is a metric that depends on the pre-test probability (i.e., probability of presence of disease state before the measurement) (28), the lower the prevalence of certain EDSS levels, the lower the PPV (and higher the NPV) will be.

The best performing classifier was a binary assessment of EDSS ≥6.0 vs. EDSS <6.0, resulting in a sensitivity 0.65 and a PPV of 0.85 for EDSS ≥6.0 (NPV = 0.93 and F1-score = 0.73), compared to a sensitivity of 0.98 and a PPV of 0.93 for EDSS <6.0 (NPV = 0.85 and F1-score = 0.95). Notably, the EDSS combines two distinct scales, whereby EDSS <6.0 is reflective of sign and symptoms based on the FS and EDSS ≥6.0 reflecting ambulation status. With challenges in the coding of relevant signs/symptoms in claims data, the binary classifier may be most useful in describing the overall ambulation status at population level. Our model shows a better performance compared to other models previously reported. Alves et al. (13) developed a machine learning model that estimated a numeric EDSS score at a specific encounter date based on clinician notes from the medical records. The model was able to estimate EDSS ≥6.0 with a PPV of 0.85 and NPV of 0.85 (13). In the Canadian study already discussed above, Marrie et al. (12) reported a sensitivity of 0.49, a PPV of 0.72, and a maximum Kappa coefficient of 0.55 (our binary model achieved a kappa of 0.69) for predicting an EDSS ≥6.0 (12).

It should also be noted that the application of our algorithm to the wider AOK PLUS cohort, resulted in a typically bimodal distribution of EDSS (27). Moreover, older patients, patients with progressive forms of MS and those with higher comorbidity burden showed higher pEDSS values which is consistent with the MS epidemiology (29, 30). This further reinforces the validity of our algorithm.

Our study has multiple strengths. While the eight-fold classifier performed poorly, our model using three and two category groupings of disability showed good to high predictive performance, and their practical utility was demonstrated in a large MS population. The development of our model was an interdisciplinary effort, involving clinicians, epidemiologists and data scientists with vast experience in MS and real-word research. This allowed to create a rule-based algorithm with comprehensive information on symptoms, medications, and aids. Most importantly, we followed a validation strategy using the EDSS derived by clinicians as the reference standard. Several studies have previously developed algorithms to assign MS disability levels based on observable claims data or EHR data sources, however a formal validation was not possible due to lack of true EDSS measures (6–8, 10, 11, 31–33).

We acknowledge some limitations. The validation cohort included only 100 patients, with each patient able to contribute multiple EDSS measurements across the follow-up period (620 measures in total). Patients were insured by AOK PLUS in the regions of Saxony/Thuringia and receiving care at a single MS center. While the results are likely generalizable to Germany, given that uniform healthcare regulations and standard clinical practices are imposed nationally, they may not be generalizable to other countries. There was also an imbalance in the true EDSS distribution, with a bias toward lower EDSS levels (1.5 to 4.0) and fewer patients with EDSS ≥7.0. A number of factors contributed to this, namely slow recruitment rates (patients were recruited based on regularly scheduled visits at ZKN), mismatch of data coverage timelines between the two datasets, and data linkage issues. It is possible that the performance of the algorithm would decrease if the population had different characteristics. However, the binary model that separated the cohort into patients with EDSS <6.0 and EDSS ≥6.0 had excellent precision and sensitivity which suggests that the algorithm is robust. It should also be noted that, as data used in this study come from standard clinical practice, there may be a small degree of miscoding, missing, or incorrect entries. Despite this, claims data are a valid source of real-world evidence and systems are in place to ensure quality of MSDS^3D^ data entries. Finally, we followed a rule-based approach informed by clinical input to develop our algorithm, an approach that could be biased. Machine learning models, and other more sophisticated deep learning-based natural language processing methods are promising alternatives (13, 15). However, as discussed above, the performance of these models was overall inferior to our algorithm, which indicates that further work is required. Insurance claims are also probably unsuitable to estimate low EDSS scores as the relevant information will likely be recorded in the clinical notes in the EHR. A combination of rule-based and machine-learning models using data from both insurance claims and the medical notes is likely to yield the best results, and we recommend that this should be an area of active research.

## 5. Conclusion

In summary, we developed and validated a rule-based proxy EDSS algorithm for estimating disability status using claims data, with a model for two and three EDSS categories showing good-to-high performance. We highlight the need for creating and maintaining linked databases such as the one used for this validation study to leverage the strengths of different real-world sources. Our study is another step forward in quantifying the level of disability and disease progression among real-world patients with MS observed in claims databases, and in turn improving real-world evidence research in MS.

## Data availability statement

The datasets presented in this article are not readily available because of ethical and privacy restrictions. Requests to access the datasets should be directed to the corresponding author.

## Ethics statement

The studies involving humans were approved by the Ethics Committee of the University of Dresden and the Ministry of Saxonia (SGB § 75). Patient written informed consent was obtained from all patients whose retrospective data was used for validation prior to linking patient data from MSDS^3D^ to claims records from AOK PLUS. The studies were conducted in accordance with the local legislation and institutional requirements. Written informed consent for participation among patients described in the broader AOK PLUS cohort was not required from the participants or the participants' legal guardians/next of kin in accordance with the national legislation and institutional requirements.

## Author contributions

EM-L: Conceptualization, Methodology, Supervision, Writing—original draft, Writing—review and editing. MG: Data curation, Formal analysis, Methodology, Project administration, Supervision, Writing—original draft, Writing—review and editing. EZ: Formal analysis, Methodology, Project administration, Writing—original draft, Writing—review and editing. AD: Methodology, Resources, Writing—review and editing. UM: Methodology, Resources, Writing—review and editing. TW: Methodology, Supervision, Writing—review and editing. TZ: Methodology, Resources, Supervision, Writing—review and editing. LC: Conceptualization, Methodology, Supervision, Writing—original draft, Writing—review and editing.
