# Supplementary material for: Claims-based algorithm to estimate the Expanded Disability Status Scale for multiple sclerosis in a German health insurance fund: a validation study using patient medical records
Source: Front Neurol. 2023 Dec 7;14:1253557. doi: 10.3389/fneur.2023.1253557 (PMC10734797; doi:10.3389/fneur.2023.1253557)
Supplement: Supplementary file 1 [file Data_Sheet_1.docx]

Supplementary Material

Claims-based algorithm to estimate the Expanded Disability Status Scale for multiple sclerosis in a German health insurance fund: a validation study using patient medical records

Erwan Muros-Le Rouzic^1†^, Marco Ghiani^2†,^ Evi Zhuleku^3*,^ Anja Dillenseger^4^, Ulf Maywald^5^, Thomas Wilke^2^, Tjalf Ziemssen^4+^, Licinio Craveiro^1+^

*** Correspondence:** Evi Zhuleku, evi.zhuleku@cytel.com

# Supplementary Figures and Tables

## Supplementary Figures

##
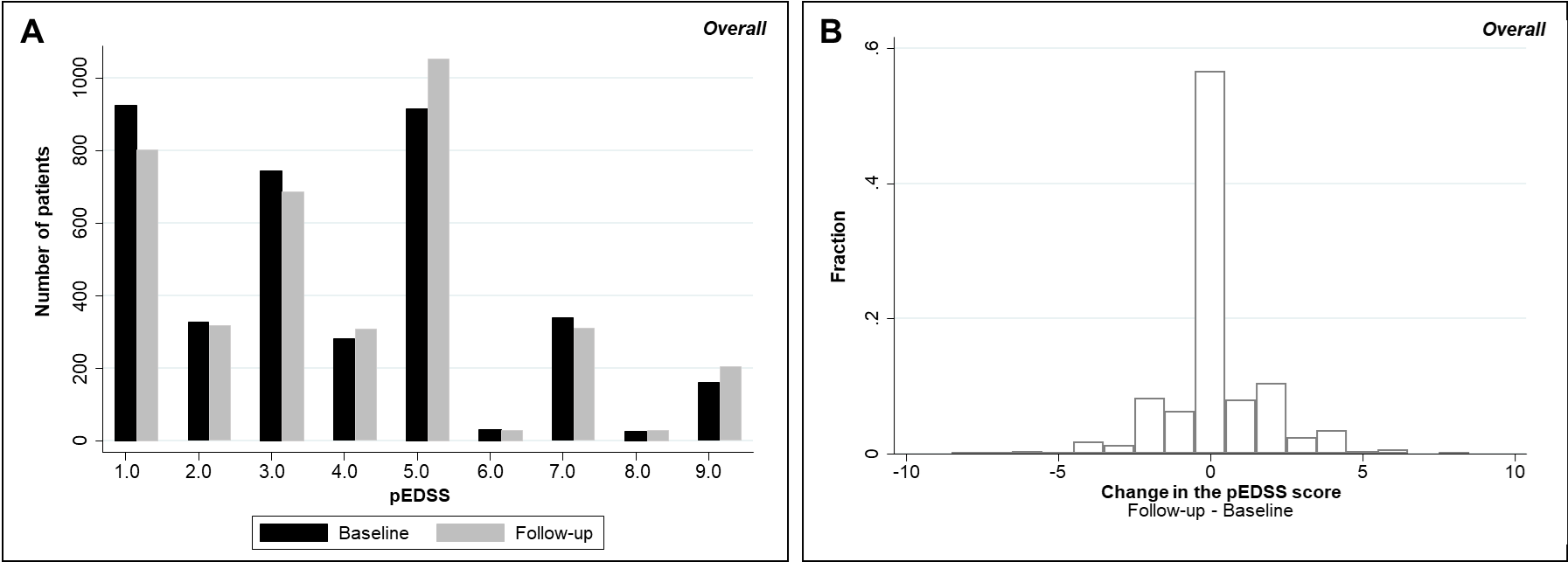


## Supplementary Figure 1. Distribution of pEDSS at baseline and follow-up among the overall AOK PLUS MS cohort (A) and respective distribution of change in the pEDSS (B).


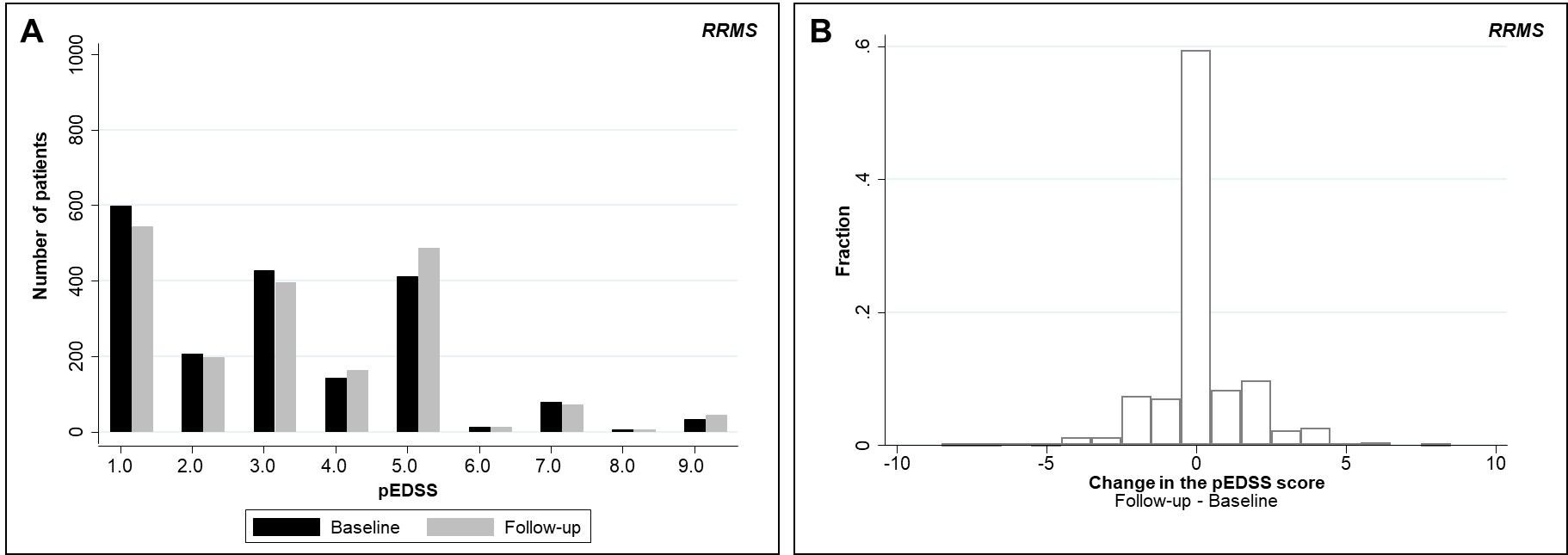


**Supplementary Figure 2.** Distribution of pEDSS at baseline and follow-up among the overall AOK PLUS RRMS cohort (A) and respective distribution of change in the pEDSS (B).


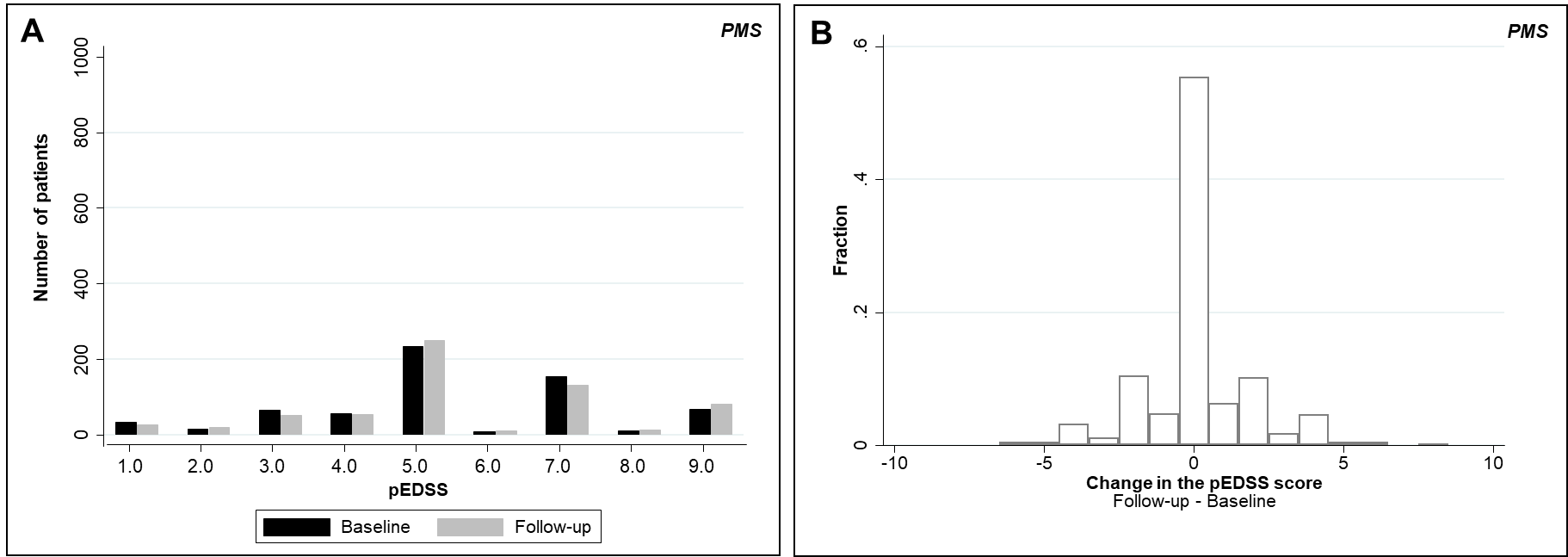


**Supplementary Figure 3.** Distribution of pEDSS at baseline and follow-up among the overall AOK PLUS PMS cohort (A) and respective distribution of change in the pEDSS (B).

## Supplementary Tables

**Supplementary Table 1. MS-related symptoms definitions and severity per functional system for computation of EDSS proxy**

| ICD-10-GM | Description | Severity | Patients with Diagnosis (N) | Comment |
| --- | --- | --- | --- | --- |
| **Cerebral** |  |  |  |  |
| R53 | Malaise and fatigue and unspecified debility | Mild | 2 | Although fatigue and depression have a high impact in QoL, the cerebral FSS has a reduced contribution to the EDSS calculation. Therefore, all these items are categorized as mild. |
| G93.3 | Chronic fatigue syndrome | Mild | 3 |  |
| F32 / F33 | Depressive disorder not elsewhere classified | Mild | 27 |  |
| F02.8 | Dementia | Severe | 0 |  |
| **Visual** |  |  |  |  |
| H54.3 | Mild visual impairment | Mild | 0 |  |
| H54.2 / H54.6 / H47.5 / H47.6 / H47.7 / H53.4 | Moderate visual impairment | Moderate | 3 |  |
| H54.1/54.5 | Severe visual impairment | Severe | 0 |  |
| H54.0/54.4 | Diagnosis of Blindness | Severe | 3 |  |
| **Sensory** |  |  |  |  |
| R20.2 / R20.3 / R20.8 | Mild disturbance of skin sensation | Mild | 3 | **Mild: i**f ICD-10-GM: R20.8 is present alone  **Moderate**: If ICD-10-GM: R20.8 is combined with gait abnormalities (R26.0 / R26.1 / R26.2 / R26.8) |
| M54.1 / M79.2 | Neuralgia neuritis and radiculitis unspecified | Mild to moderate | 9 | **Mild pain**: Prescription of medications limited to paracetamol (ATC: N02BE01) and/or NSAIDs (ATC: M01A)  **Moderate pain**: Prescription of opioids (ATC: N02A), or anticonvulsants (ATC: N03A), or metamizole (ATC: N02BB02) |
| M79.6 | Pain in limb | Mild to moderate | 3 |  |
| R52 | Generalized pain | Mild to moderate | 8 |  |
| M54.2 | Cervicalgia | Mild to moderate | 8 |  |
| R52.1 / R52.2 / F45.21 | Chronic pain | Mild to moderate | 8 |  |
| G50.0 | Trigeminal neuralgia | Mild to moderate | 2 |  |
| R20.1 | Hypoesthesia of the skin | Moderate | 4 |  |
| R20.0 | Anaesthesia of the skin | Severe | 0 |  |
| **Bowel/Bladder** |  |  |  |  |
| R32 | Urinary incontinence unspecified | Mild to severe | 9 | **Mild**: if clinical descriptor is present but no medication or other treatment  **Moderate (EDSS 3 or 4):** pharmacological treatment is used:   - **Antimuscarinic agents:** All drugs in the ATC class G04BD - **Desmopressin** (ATC: H01BA02) - **Alfa 1-blockers:** terazosin (ATC: G04CA03), doxazosin (ATC: C02CA04), tamsulosin (ATC: G04CA02), and alfuzosin (ATC: G04CA01) - **Muscle relaxants:** Baclofen (ATC: M03BX01) and Dantrolen (ATC: M03CA01)   **Severe (leading to EDSS 4 or 5)**: if clinical descriptor is present together with prescription of one of the following:   - Intravesical Botox (ATC: M03AX01) OR - Root stimulation (OPS: 5-039.8, 5-039.g, 5-039.j, 5-039.p, 5-039.k) OR - Self-catheterisation (OPS: 8-123 / 8-124 / 8-128 / 8-133 / 8-137 / 8-138 / 8-139) OR - Bladder augmentation (OPS: 5-578.6) - Cystectomy (OPS: 5-576) - Continent and incontinent urinary diversion (OPS: 5-564, 5-565, 5-566, 5-567) |
| R30.1 | Tenesmus vesicae | Mild to severe | 0 |  |
| R33 | Urinary retention | Mild to severe | 2 |  |
| R35 | Polyuria, including frequent voiding | Mild to severe | 0 |  |
| R39.1 | Other micturition disorders | Mild to severe | 6 |  |
| N39.3 | Stress incontinence | Mild to severe | 2 |  |
| N39.4 | Other urinary incontinence (including urge incontinence and overflow incontinence) | Mild to severe | 6 |  |
| N39.9 | Other functional disorders of bladder | Mild to severe | 0 |  |
| N32.8 | Other specified diseases of the bladder (Including: calcified, contracted and overactive bladder) | Mild to severe | 0 |  |
| N31.0 | Uninhibited neurogenic bladder emptying, unclassified elsewhere | Mild to severe | 1 |  |
| N31.1 | Neurogenic reflex bladder, not elsewhere classified | Mild to severe | 3 |  |
| N31.88 | Other neuromuscular dysfunction of the bladder | Mild to severe | 2 |  |
| N32.9 | Diseases of the bladder, unspecified | Mild to severe | 1 |  |
| N31.9 | Neuromuscular dysfunction of the bladder, unspecified. | Mild to severe | 4 |  |
| K59.0 | Constipation | Mild to severe | 3 | **Mild**: if constipation only  **Moderate**: if any of the clinical descriptors and the following treatments:   - Bulk-forming: methylcellulose (ATC: A06AC06), ispaghula (ATC: A06AC01), ethulose (ATC: A06AC02), sterculia (A06AC03), linseed (ATC: A06AC05), triticum (ATC: A06AC07), polycarbophil calcium (ATC: A06AC08) - Stimulant: bisacodyl (ATC: A06AB02), senna (ATC: A06AB06) - Osmotic laxatives: lactulose (ATC: A06AD11) - Faecal softeners: docusate sodium (ATC: A06AA02), prucalopride (ATC: A06AX05), linaclotide (ATC: A06AX04) - Enema (OPS: 8-121procedure)   **Severe**: if any of the clinical descriptors and the following treatments:   - Transanal irrigation (OPS: 8-126) - Sacral anterior nerve root stimulation or neuromodulation - Tibial nerve stimulation - Colostomy (OPS: 5-463.2) /Ileostomy (OPS: 5-460 / 5-461 / 5-462) |
| K59.2 | Neurogenic bowel, not elsewhere classified | Mild to severe | 1 |  |
| R15 | Faecal incontinence | Severe | 2 |  |
| **Pyramidal** |  |  |  |  |
| R25/G95 | Spasticity | Mild to severe | 4 | **Mild spasticity (EDSS 1 or 2):** only clinical descriptor  **Moderate spasticity (EDSS 3 or 4):** clinical descriptor + pharmacological treatment:   - Baclofen (ATC: M03BX01) - Dantrolene (ATC: M03CA01) - Tizanidine (ATC: M03BX02) - Clonazepam (ATC: N03AE) - Diazepam (ATC: N05BA01) - Alprazolam (ATC: N05BA12) - Lorazepam (ATC: N05BA06)   **Severe spasticity (EDSS 5):** clinical descriptor + interventional treatment:   - Intrathecal baclofen pump (EBM code: 30751) - Botox (ATC: M03AX01) - Triamcinolone (ATC: H02AB08) |
| M62.4 | Spasm of muscle | Mild to severe | 0 |  |
| G82.13 | Paraparesis and paraplegia, spastic: Chronic incomplete paraplegia | Moderate | 7 | This indicates Paraparesis |
| G82.23 | Paraparesis and paraplegia, unspecified: Chronic incomplete paraplegia | Moderate | 0 | This indicates Paraparesis |
| G83.1 | Monoplegia/monoparesis of lower limb affecting dominant, non-dominant or unspecified side | Mild | 0 |  |
| G83.2 | Monoplegia/monoparesis of upper limb affecting dominant, non-dominant or unspecified side | Mild | 0 |  |
| G83.3 | Unspecified monoplegia/monoparesis | Moderate | 0 |  |
| G83.0 | Diplegia/diparesis of upper limbs | Moderate | 0 |  |
| G82.43 | Tetraparesis and tetraplegia, spastic: Chronic incomplete spinal cord injury | Severe | 3 | This indicates Tetraparesis |
| M62.5 | Muscle wasting and atrophy, not elsewhere classified | Severe | 0 |  |
| R26.1 / R26.2 / R26.8 | Abnormality of gait, difficulty walking | **Severe** | 12 |  |
| G82.53 | Tetraparesis and tetraplegia, unspecified: Chronic incomplete spinal cord injury | Severe | 0 | This indicates Tetraparesis |
| G83.9 | Paralytic syndrome unspecified | Severe | 1 |  |
| G81.1 | Spastic hemiplegia and hemiparesis | Severe | 4 |  |
| G81.9 | Hemiplegia and hemiparesis, unspecified | Severe | 2 |  |
| M62.3 | Immobility syndrome (paraplegic) | EDSS 7.0 | 1 | This indicates Paraplegia |
| G82.12 | Paraparesis and Paraplegia, spastic: Chronic Complete Paraplegia | EDSS 7.0 | 0 | This indicates Paraplegia |
| G82.22 | Paraparesis and paraplegia, unspecified: Chronic complete spinal cord injury | EDSS 7.0 | 1 | This indicates Paraplegia |
| G82.42 | Tetraparesis and tetraplegia, spastic: Chronic complete spinal cord injury | EDSS 8.0 | 1 | This indicates Tetraplegia |
| G82.52 | Tetraparesis and tetraplegia, unspecified: Chronic complete spinal cord injury | EDSS 8.0 | 0 | This indicates Tetraplegia |
| **Cerebellar** |  |  |  |  |
| R27.0 / R27.8 | Lack of coordination | Mild | 1 | **Mild tremor (EDSS 1 or 2):** only clinical descriptor  **Moderate tremor (EDSS 3 or 4):** clinical descriptor + pharmacological treatment:   - Topiramate (ATC: N03AX11) - Riluzole (ATC: N07XX02) - Isoniazid (ATC: J04AC01) - Carbamazepine (ATC: N03AF01) - Glutethimide (ATC: N05CE01) - Levetiracetam (ATC: N03AX14) - Propranolol (ATC: C07AA05) - Ondansetron (ATC: A04AA01)   **Severe tremor (EDSS 5):** clinical descriptor + interventional treatment:   - Intrathecal baclofen (EBM code: 30751) - Botox (ATC: M03AX01) - Deep brain stimulation (OPS: 8-631) - Thalamotomy (OPS: 5-013.72) |
| G32.8 | Cerebellar ataxia in diseases classified elsewhere | Mild | 0 |  |
| G25.0 / G25.1 / G25.2 | Essential and other specified forms of tremor | Mild to severe | 0 |  |
| R26.0 | Ataxic gait | Moderate | 2 |  |
| R29.6 | Tendency to fall, not elsewhere classified | Moderate | 2 |  |
| **Brainstem** |  |  |  |  |
| H51 | Other disorders of binocular movement | Severe | 0 |  |
| H49.0 / H49.1 / H49.2 / H49.9 | Paralytic strabismus of oculomotor nerve / trochlear nerve / abducens nerve / unspecified | Mild | 0 |  |
| H90.3 / H90.4 / H90.5 | Sensorineural hearing loss | Mild | 0 |  |
| H81.2 / H81.4 / H81.8 / H81.9 | Disorders of vestibular function | Moderate | 1 |  |
| H82 | Vertigo syndromes in diseases classified elsewhere | Moderate | 0 |  |
| G83.6 | Central facial paresis | Moderate | 0 |  |
| H53.2 | Diplopia | Moderate | 1 |  |
| H55 | Nystagmus | Moderate | 0 |  |
| R47.1 | Dysarthria | Moderate to severe | 0 | **Moderate:** if clinical descriptor only  **Severe:** if clinical descriptor and treatment:   - **Dysphagia**: - Pharyngeal stimulation therapy (OPS: 8-633) - Brain neurostimulation (OPS: 8-631.0) - **Dysarthria**: - Speech therapy (therapy code SP) OR - Brain neurostimulation (OPS: 8-631.0) |
| R13 | Dysphagia | Moderate to severe | 0 |  |

***Abbreviations:*** *ATC, Anatomical Therapeutic Chemical Classification; EDSS, Expanded Disability Status Scale; FSS, Functional system score; ICD-10-GM, International Classification of Diseases 10, German Modification; OPS, Operations and Procedures Coding System; QoL, Quality of life*

Supplementary Table 2. Functional system sub-scores in MSDS^3D^ by symptoms and their severity observed in claims data

| ****Functional System**** | ****MSDS^3D^**** | ****AOK PLUS**** | | | | |
| --- | --- | --- | --- | --- | --- | --- |
|  | **True FSS** | **No symptoms** | **≥1 symptom (any severity)** | **Mild symptoms** | **Moderate symptoms** | **Severe symptoms** |
| ****Visual**** | **N = 100** | **N = 94** | **N = 6** | **N = 0** | **N = 3** | **N = 3** |
| 0. Normal | 36 (36.0) | 36 (38.3) | - | - | - | - |
| 1. Disc pallor and/or small scotoma and/or visual acuity of worse eye less than 20/20 (1.0) but better than 20/30 (0.67) | 28 (28.0) | 26 (27.7) | 2 (33.3) | - | 1 (33.3) | 1 (33.3) |
| 2. Worse eye with maximal visual acuity of 20/30 to 20/59 (0.67–0.34) | 24 (24.0) | 23 (24.5) | 1 (16.7) | - | 1 (33.3) | - |
| 3. Worse eye with large scotoma and/or moderate decrease in fields and/or maximal visual acuity of 20/60 to 20/99 (0.33–0.21) | 9 (9.0) | 6 (6.4) | 3 (50.0) | - | 1 (33.3) | 2 (66.7) |
| 4. Worse eye with marked decrease of fields and/or maximal visual acuity of 20/100 to 20/200 (0.2–0.1); Grade 3 plus maximal acuity of better eye of 20/60 (0.33) or less | 1 (1.0) | 1 (1.1) | - | - | - | - |
| 5. Worse eye with maximal visual acuity less than 20/200 (0.1); Grade 4 plus maximal acuity of better eye of 20/60 (0.33) or less | 0 (0.0) | - | - | - | - | - |
| 6. Grade 5 plus maximal visual acuity of better eye of 20/60 (0.33) or less | 0 (0.0) | - | - | - | - | - |
| Missing | 2 (2.0) | 2 (2.1) | - | - | - | - |
| Brainstem | **N = 100** | **N = 98** | **N = 2** | **NA** | **N = 2** | **N = 0** |
| 0. Normal | 30 (30.0) | 30 (30.6) | - | NA | - | - |
| 1. Signs only | 50 (50.0) | 49 (50.0) | 1 (50.0) | NA | 1 (50.0) | - |
| 2. Moderate nystagmus and/or moderate extraocular movements impairment and/or other mild disability | 16 (16.0) | 15 (15.3) | 1 (50.0) | NA | 1 (50.0) | - |
| 3. Severe nystagmus and/or marked extraocular movements impairment weakness and/or moderate disability of other cranial nerves | 3 (3.0) | 3 (3.1) | - | NA | - | - |
| 4. Marked dysarthria and/or marked disability | 0 (0.0) | - | - | NA | - | - |
| 5. Inability to swallow or speak | 0 (0.0) | - | - | NA | - | - |
| Missing | 1 (0.0) | 1 (1.0) | - | NA | - | - |
| Pyramidal | **N = 100** | **N = 77** | **N = 23** | **N = 0** | **N = 10** | **N = 18** |
| 0. Normal | 8 (8.0) | 8 (10.4) | - | - | - | - |
| 1. Abnormal signs without disability | 32 (32.0) | 31 (40.3) | 1 (4.4) | - | - | 1 (5.6) |
| 2. Minimal disability | 30 (30.0) | 23 (29.9) | 7 (30.4) | - | 5 (50.0) | 3 (16.7) |
| 3. Mild to moderate paraparesis or hemiparesis | 25 (25.0) | 13 (16.9) | 12 (52.2) | - | 5 (50.0) | 11 (61.1) |
| 4. Marked paraparesis or hemiparesis | 3 (3.0) | 1 (1.3) | 2 (8.7) | - | - | 2 (11.1) |
| 5. Paraplegia or hemiplegia | 1 (1.0) | - | 1 (4.4) | - | - | 1 (5.6) |
| 6. Tetraplegia | 0 (0.0) | - | - | - | - | - |
| Missing | 1 (1.0) | 1 (1.3) | - | - | - | - |
| Cerebellar | **N = 100** | **N = 96** | **N = 4** | **N = 1** | **N = 3** | **N = 0** |
| 0. Normal | 23 (23.0) | 23 (24.0) | - | - | - | - |
| 1. Abnormal findings but no disability | 38 (38.0) | 36 (37.5) | 2 (50.0) | 1 (100.0) | 1 (33.3) | - |
| 2. Mild ataxia and/or moderate station ataxia | 20 (20.0) | 19 (19.8) | 1 (25.0) | - | 1 (33.3) | - |
| 3. Moderate truncal ataxia and/or moderate limb ataxia and/or moderate or severe gait/truncal ataxia | 16 (16.0) | 16 (16.7) | - | - | - | - |
| 4. Severe gait/truncal ataxia and severe ataxia in three or four limbs | 2 (2.0) | 1 (1.0) | 1 (25.0) | - | 1 (33.3) | - |
| 5. Unable to perform coordinated movements related to ataxia | 0 (0.0) | - | - | - | - | - |
| Missing | 1 (1.0) | 1 (1.0) | - | - | - | - |
| Sensory | **N = 100** | **N = 75** | **N = 25** | **N = 10** | **N = 16** | **NA** |
| 0. Normal | 19 (19.0) | 15 (20.0) | 4 (16.0) | 2 (20.0) | 2 (12.5) | NA |
| 1. Vibration or figure-writing decrease only in one or two limbs | 27 (27.0) | 22 (29.3) | 5 (20.0) | 3 (30.0) | 3 (18.8) | NA |
| 2. Mild decrease in touch, pain or position sense, and/or moderate decrease in vibration in 1 or 2 limbs; or vibratory decrease alone in 3 or 4 limbs | 31 (31.0) | 22 (29.3) | 9 (36.0) | 3 (30.0) | 6 (37.5) | NA |
| 3. Moderate decrease in touch or pain or position sense, and/or essentially lost vibration in 1 or 2 limbs; or mild decrease in touch or pain and/or moderate decrease in all proprioceptive tests in 3 or 4 limbs | 20 (20.0) | 14 (18.7) | 6 (24.0) | 2 (20.0) | 4 (25.0) | NA |
| 4. Marked decrease in touch or pain or loss of proprioception, alone or combined, in 1 or 2 limbs; or moderate decrease in touch or pain and/or severe proprioceptive decrease in more than 2 limbs | 2 (2.0) | 1 (1.3) | 1 (4.0) | - | 1 (6.3) | NA |
| 5. Loss (essentially) of sensation in 1 or 2 limbs; or moderate decrease in touch or pain and/or loss of proprioception for most of the body below the head | 0 (0.0) | - | - | - | - | NA |
| 6. Sensation essentially lost below the head | 0 (0.0) | - | - | - | - | NA |
| Missing | 1 (1.0) | 1 (1.33) | - | - | - | NA |
| Bowel and bladder | **N = 100** | **N = 76** | **N = 24** | **N = 5** | **N = 14** | **N = 2** |
| 0. Normal | 43 (43.0) | 40 (52.6) | 3 (12.5) | 2 (40.0) | - | - |
| 1. Mild urinary hesitance, urgency, or retention | 35 (35.0) | 26 (34.2) | 9 (37.5) | 1 (20.0) | 5 (35.7) | 1 (50.0) |
| 2. Moderate hesitance, urgency, retention of bowel/bladder, or rare urinary incontinence | 13 (13.0) | 7 (9.2) | 6 (25.0) | 2 (40.0) | 3 (21.4) | 1 (50.0) |
| 3. Frequent urinary incontinence | 6 (6.0) | 2 (2.6) | 4 (16.7) | - | 4 (28.6) | - |
| 4. In need of almost constant catheterization | 2 (2.0) | - | 2 (8.3) | - | 2 (14.3) | - |
| 5. Loss of bladder function | 0 (0.0) | - | - | - | - | - |
| 6. Loss of bowel and bladder function | 0 (0.0) | - | - | - | - | - |
| Missing | 1 (1.0) | 1 (1.32) | - | - | - | - |
| Cerebral | **N = 100** | **N = 73** | **N = 27** | **N = 27** | **NA** | **N = 0** |
| 0. Normal | 29 (29.0) | 26 (35.6) | 3 (11.1) | 3 (11.1) | NA | - |
| 1. Mood alteration only (no effect on EDSS score) | 30 (30.0) | 22 (30.1) | 8 (29.6) | 8 (29.6) | NA | - |
| 2. Mild decrease in mentation | 39 (39.0) | 23 (31.5) | 16 (59.3) | 16 (59.3) | NA | - |
| 3. Moderate decrease in mentation | 1 (1.0) | 1 (1.4) | - | - | NA | - |
| 4. Marked decrease in mentation | 0 (0.0) | - | - | - | NA | - |
| 5. Dementia | 0 (0.0) | - | - | - | NA | - |
| Missing | 1 (1.0) | 1 (1.4) | - | - | NA | - |
| ****Ambulation**** | **True FSS** | **No aids** | **Bed confined** | **Walking stick** | **Specialty bed** | **Wheelchair** |
|  | **N = 100** | **N = 87** | **N = 0** | **N = 4** | **N = 1** | **N = 10** |
| 0. Unrestricted | 48 (48.0) | 48 (55.2) | - | - | - | - |
| 1. Fully ambulatory | 26 (26.0) | 24 (27.6) | - | 1 (25.0) | - | 1 (10.0) |
| 2. >300 meters, <500 meters, w/o help or assistance (EDSS 4.5 or 5.0) | 4 (4.0) | 4 (4.6) | - | - | - | - |
| 3. >200 meters, but <300 meters, w/o help or assistance (EDSS 5.0) | 1 (1.0) | 1 (1.2) | - | - | - | - |
| 4. >100 meters, but <200 meters, w/o help or assistance (EDSS 5.5) | 2 (2.0) | 2 (2.3) | - | - | - | - |
| 5. Walking range <100 meters without assistance (EDSS 6.0) | 0 (0.0) | - | - | - | - | - |
| 6. Unilateral assistance, >50 meters (EDSS 6.0) | 1 (1.0) | 1 (1.2) | - | - | - | - |
| 7. Bilateral assistance, >120 meters (EDSS 6.0) | 3 (3.0) | 2 (2.3) | - | 1 (25.0) | - | - |
| 8. Unilateral assistance, <50 meters (EDSS 6.5) | 1 (1.0) | - | - | 1 (25.0) | - | - |
| 9. Bilateral assistance, >5 meters, but <120 meters (EDSS 6.5) | 4 (4.0) | 2 (2.3) | - | - | - | 2 (20.0) |
| 10. Uses wheelchair w/o help; unable to walk 5 meters even with aid, essentially restricted to wheelchair; wheels self and transfers alone; up and about in wheelchair some 12 hours a day (EDSS 7.0) | 5 (5.0) | - | - | 1 (25.0) | - | 5 (50.0) |
| 11.Uses wheelchair with help; unable to take more than a few steps; restricted to wheelchair; may need some help in transfer/wheeling self (EDSS 7.5) | 2 (2.0) | 1 (1.2) | - | - | - | 1 (10.0) |
| 12.Restricted to bed or chair or perambulated in wheelchair/out of bed most of day/retains many self-care functions; effective use of arms (EDSS 8.0) | 0 (0.0) | - | - | - | - | - |
| Missing | 3 (3.0) | 2 (2.3) | - | - | 1 (100.0) | 1 (10.0) |

***Abbreviations:*** *EDSS, Expanded Disability Status Scale; FSS, functional system score*

Supplementary Table 3. Predictive performance of EDSS proxy as eight-fold EDSS classifier, three-fold, and binary severity classifier of all EDSS in the follow-up

|  | ****TP**** | ****TN**** | ****FP**** | ****FN**** | ****Sensitivity****  ****(Recall)**** | ****Specificity**** | ****PPV****  ****(Precision)**** | ****NPV**** | ****Accuracy**** | ****Cohen’s Kappa**** | ****F1-Score**** |
| --- | --- | --- | --- | --- | --- | --- | --- | --- | --- | --- | --- |
| ****Eight-fold EDSS classifier**** | | | | | | | | | | | |
| **EDSS 1.0** | 78 | 319 | 198 | 25 | 0.76 | 0.62 | 0.28 | 0.93 | 0.64 | 0.22 | 0.41 |
| **EDSS 2.0** | 20 | 438 | 22 | 140 | 0.13 | 0.95 | 0.48 | 0.76 | 0.74 | 0.10 | 0.20 |
| **EDSS 3.0** | 42 | 363 | 89 | 126 | 0.25 | 0.80 | 0.32 | 0.74 | 0.65 | 0.06 | 0.28 |
| **EDSS 4.0** | 11 | 499 | 26 | 84 | 0.12 | 0.95 | 0.30 | 0.86 | 0.82 | 0.09 | 0.17 |
| **EDSS 5.0** | 7 | 535 | 69 | 9 | 0.44 | 0.89 | 0.09 | 0.98 | 0.87 | 0.11 | 0.15 |
| **EDSS 6.0** | 10 | 547 | 9 | 54 | 0.16 | 0.98 | 0.53 | 0.91 | 0.90 | 0.20 | 0.24 |
| **EDSS 7.0** | 6 | 580 | 31 | 3 | 0.67 | 0.95 | 0.16 | 0.99 | 0.95 | 0.24 | 0.26 |
| **EDSS ≥8.0** | 0 | 613 | 2 | 5 | 0.00 | 1.00 | 0.00 | 0.99 | 0.99 | 0.00 | 0.00 |
| ****Three-fold classifier**** | | | | | | | | | | | |
| **EDSS 1.0-3.0** | 371 | 111 | 78 | 60 | 0.86 | 0.59 | 0.83 | 0.65 | 0.78 | 0.46 | 0.84 |
| **EDSS 4.0-5.0** | 38 | 434 | 75 | 73 | 0.34 | 0.85 | 0.34 | 0.86 | 0.76 | 0.19 | 0.34 |
| **EDSS ≥6.0** | 44 | 528 | 14 | 34 | 0.56 | 0.97 | 0.76 | 0.94 | 0.92 | 0.60 | 0.65 |
| ****Binary classifier**** | | | | | | | | | | | |
| **EDSS <6.0** | 528 | 44 | 34 | 14 | 0.97 | 0.56 | 0.94 | 0.76 | 0.92 | 0.60 | 0.96 |
| **EDSS ≥6.0** | 44 | 528 | 14 | 34 | 0.56 | 0.97 | 0.76 | 0.94 | 0.92 | 0.60 | 0.65 |

**Sensitivity/Recall: TP / (TP+FN); Specificity: TN / (FP+TN); PPV/Precision: TP / (TP+FP); NPV: TN / (FN+TN); Accuracy: (TP+TN) / (TP+TN+FP+FN); F1-Score: harmonic mean of recall and precision**

***Abbreviations:*** *EDSS, Expanded Disability Status Scale; FN, false negative; FP, false positive; NPV, negative prediction value; PPV, positive prediction value; TN, true negative; TP, true positive*

Supplementary Table 4. pEDSS of the AOK PLUS cohort in the baseline according to key patient characteristics

| Category | | N | Overall pEDSS | pEDSS, n (%) | | | pEDSS, n (%) | |
| --- | --- | --- | --- | --- | --- | --- | --- | --- |
|  |  |  | **Mean (SD)** | **0 - 3.0** | **4.0 - 5.0** | **≥6.0** | **<6.0** | **≥6.0** |
| Overall | All | 3756 | 3.7 (2.2) | 1995 (53.1) | 1198 (31.9) | 563 (15.0) | 3193 (85.0) | 563 (15.0) |
| Sex | Female | 2700 | 3.6 (2.2) | 1477 (54.7) | 854 (31.6) | 369 (13.7) | 2331 (86.3) | 369 (13.7) |
|  | Male | 1056 | 3.9 (2.3) | 518 (49.1) | 344 (32.6) | 194 (18.4) | 862 (81.6) | 194 (18.4) |
| Age (years) | 18-50 | 1716 | 2.7 (1.9) | 1248 (72.7) | 366 (21.3) | 102 (5.9) | 1614 (94.1) | 102 (5.9) |
|  | 51-65 | 1357 | 4.1 (2.1) | 589 (43.4) | 542 (39.9) | 226 (16.7) | 1131 (83.3) | 226 (16.7) |
|  | >65 | 683 | 5.3 (2.2) | 158 (23.1) | 290 (42.5) | 235 (34.4) | 448 (65.6) | 235 (34.4) |
| Type of MS | RRMS | 1929 | 3.1 (1.9) | 1237 (64.1) | 557 (28.9) | 135 (7.0) | 1794 (93.0) | 135 (7.0) |
|  | PMS | 652 | 5.4 (2.1) | 118 (18.1) | 292 (44.8) | 242 (37.1) | 410 (62.9) | 242 (37.1) |
|  | Unspecified | 1175 | 3.7 (2.3) | 640 (54.5) | 349 (29.7) | 186 (15.8) | 989 (84.2) | 186 (15.8) |
| Baseline  comorbidities | 0 | 1056 | 2.4 (1.8) | 827 (78.3) | 183 (17.3) | 46 (4.4) | 1010 (95.6) | 46 (4.4) |
|  | 1 | 1019 | 3.4 (2.0) | 587 (57.6) | 331 (32.5) | 101 (9.9) | 918 (90.1) | 101 (9.9) |
|  | **≥2** | 1681 | 4.6 (2.2) | 581 (34.6) | 684 (40.7) | 416 (24.7) | 1265 (75.3) | 416 (24.7) |
